# Multi-Transcriptomic Analysis Reveals GSC-Driven MES-Like Differentiation via EMT in GBM Cell–Cell Communication

**DOI:** 10.3390/biomedicines13061304

**Published:** 2025-05-26

**Authors:** Weichi Wu, Po Zhang, Dongsheng Li, Kejun He

**Affiliations:** 1 Department of Neurosurgery, The First Affiliated Hospital of Sun Yat-Sen University, Guangzhou 510080, China; wuweichi@hotmail.com; 2 Department of Neurosurgery, Union Hospital, Tongji Medical College, Huazhong University of Science and Technology, Wuhan 430022, China; zhangpo1993@gmail.com; 3 Department of Emergency Medicine, The Affiliated Changsha Hospital of Xiangya School of Medicine, Central South University (The First Hospital of Changsha), Changsha 410013, China; lds15111360043@163.com

**Keywords:** glioma stem cells, epithelial–mesenchymal transition, glioblastoma, cell–cell communication

## Abstract

**Background**: Glioblastoma (GBM) is the most malignant brain tumor, with a cellular hierarchy dominated by glioma stem cells (GSCs). Understanding global communications among GSCs and other cells helps us identify potential new therapeutic targets. In this study, multi-transcriptomic analysis was utilized to explore the communication pattern of GSCs in GBM. **Methods**: CellChat was used to quantitatively infer and analyze intercellular communication networks from GBM single-cell RNA-sequencing (scRNA-seq) data. Gene set enrichment analysis (GSEA) was conducted to identify specific biological pathways (epithelial–mesenchymal transition, EMT) involved in the communication pattern of GSCs. Spatial transcriptomic database was used to support the relationship between EMT and GSC proliferation. Single-sample GSEA (ssGSEA) was employed to assess which GSC state exhibited the strongest association with the EMT signature. **Results**: The cell communication pattern of GSCs is mostly related to EMT. Multiple EMT-related genes are highly expressed in GBM, particularly in GSCs, which are associated with poor prognosis. In addition, EMT-related genes are most enriched in mesenchymal-like (MES-like) GSCs. Tumor patients with MES-like GSC-enriched signatures demonstrate the most unfavorable prognosis compared to those harboring proneural-like (PN-like) or classical-like (CL-like) GSCs. **Conclusions**: This study suggests that GSCs facilitate GBM progression through intercellular communication in the pattern of EMT. EMT-associated genes may drive the differentiation of GSCs toward a MES-like phenotype, thereby leading to poorer clinical outcomes. Consequently, targeting EMT-related pathways could represent a novel therapeutic strategy for GBM treatment.

## 1. Background

Glioblastoma (GBM) is the predominant malignant and most lethal tumor of the central nervous system (CNS), carrying the highest mortality rates [1]. Epidemiological studies indicate that the incidence rate of GBM is approximately 5.26/100,000, with nearly 17,000 new diagnoses annually [2,3,4]. The current standard of care (SoC) for GBM, including maximal surgical resection with preservation of neurologic function, chemoradiation, and adjuvant chemotherapy [5], offers only limited survival benefit, and almost all patients eventually recur, in part due to the presence of glioma stem cells (GSCs) [6]. As at the apex of cellular hierarchies in GBM, the self-renewing GSCs contribute to neovascularization, immunosuppressive adaptation, infiltrative growth patterns, and multidrug resistance phenotypes during tumor progression [7,8,9,10]. Therapeutic targeting of GSCs holds significant promise for GBM management. However, developing effective GSC-targeting strategies remains challenging due to their heterogeneity, plasticity, and varied therapeutic responses [11,12,13].

Recent studies have established foundational insights into GSC heterogeneity and epithelial–mesenchymal transition (EMT). For instance, Neftel et al. [14] delineated four GSC subtypes with distinct transcriptional programs, including oligodendrocyte precursor cell-like (OPC-like), neural progenitor cell-like (NPC-like), astrocyte-like (AC-like), and mesenchymal-like (MES-like), highlighting microenvironment-driven plasticity. However, the dynamic interplay between subtype-specific transcriptional regulators and EMT effectors remains unresolved.

Cells communicate through released chemicals and surface signals to guide key decisions like starting growth, triggering self-destruct, moving locations, or developing into specific cell types [15,16,17]. The tumor microenvironment (TME) in GBM is highly complex, containing various cell types and extracellular components. Understanding how tumor cells, particularly GSCs, interact with surrounding cells could reveal key mechanisms driving GBM progression and help identify potential treatment targets. The interplay between GSCs and other GBM cells within TME is multi-dimensional, with biophysical properties integral to oncology, and proposed as a source for new strategies for GBM management.

Single-cell RNA sequencing (scRNA-seq) allows researchers to study cell differences and development patterns in great detail [18,19]. Several strategies have been recently applied to infer cell–cell communication from scRNA-seq data [20,21,22,23,24,25,26], such as CellChat [20], Single-CellSignalR [22], and NicheNet [25]. Other than scRNA-seq, recent progress in spatially resolved transcriptomic methods allows researchers to study how cells are arranged spatially in tissues [27]. Combining these spatial data with scRNA-seq results could help provide a better understanding of cellular crosstalk in GBM [28].

Therefore, our study aims to identify communication patterns of GSCs in GBM and their impact on disease progression. Through integrated analysis of multiple scRNA-seq datasets and spatial transcriptomic data, we found that GSC communication patterns are closely associated with EMT. Further analysis showed that EMT may promote increased proportions of MES-like subtypes in GSCs, which are strongly linked to poorer patient outcomes. Importantly, targeting EMT-related genes in GBM could serve as a potential therapeutic strategy for future treatment development.

## 2. Materials and Methods

### 2.1. Data Source and Processing

Data were obtained from the Gene Expression Omnibus (GEO) (https://www.ncbi.nlm.nih.gov/geo/, accessed on 16 March 2025) database at the National Center for Biotechnology Information (NCBI) in the United States. The information on the datasets is presented in Table 1.

For the GSE84465 dataset, following data normalization, we performed differential expression analysis across all cells, conducted principal component analysis (PCA), and subsequently applied clustering and UMAP dimensionality reduction. Cell annotation was performed using markers for GSCs and differentiated glioma cells (DGCs) defined by Martina et al. [29], while employing the CellMarker database (http://xteam.xbio.top/CellMarker/#, accessed on 16 March 2025) as a reference for other cell types.

For the GSE129438 dataset, the BW-format files were visualized using the Integrative Genomics Viewer (IGV) software (version 2.17.0), with genomic data aligned to the human reference genome assembly (GRCh37/hg19).

For the GSE131928 dataset, the pre-processed data provided by the authors through the GEO repository were directly employed for subsequent analytical procedures.

### 2.2. Cell–Cell Interaction Analysis

The R package CellChat V.1.6.114 was used to quantitatively infer and analyze intercellular communication networks using scRNA-seq data of GSE84465. CellChat employs a computational framework integrating network topology analysis and pattern recognition algorithms to systematically infer principal signaling input–output relationships among cellular populations.

Inference of intercellular communications: identification of differentially expressed signaling genes. To infer the cell state-specific communications, we first identified differentially expressed signaling genes across all cell groups within the scRNA-seq dataset using the Wilcoxon rank sum test with a significance level of 0.05.

### 2.3. Functional Enrichment Analysis

The clusterProfiler package (v 3.14.3) was used to perform Kyoto Encyclopedia of Genes and Genomes (KEGG) enrichment analyses on the cellular communication pattern genes of GSCs. The gene set enrichment analysis (GSEA) software (v 4.3.3) was obtained from the GSEA website (http://software.broadinstitute.org/gsea/index.jsp, accessed on 17 March 2025) and used for GSEA enrichment analysis based on the MSigDB database [30,31] (http://www.gsea-msigdb.org/gsea/downloads.jsp, accessed on 17 March 2025). Additionally, single-sample GSEA (ssGSEA) was performed to quantify the EMT-related enrichment score of various GSC subtypes within GBM.

### 2.4. Processing of GBM Spatial Transcriptome Sequencing Data

GBM spatial transcriptome sequencing data were sourced from Datadryad (https://doi.org/10.5061/dryad.h70rxwdmj) [32]. We employed SPATA software SPATA:: plotSurfaceInteractive and SPATA2 (https://github.com/theMILOlab/SPATA2, accessed on 20 March 2025) for the visualization of spatial gene expression. For spatial expression plots, either normalized and scaled gene expression values (to plot single genes) or enrichment scores of a defined gene set were used, with the 0.5 quantile of a probability distribution fitting. The x-axis and y-axis coordinates are given by the input file based on the localization to the H&E staining.

### 2.5. Cell Lines and Cell Culture

The following cell lines were utilized in this study: GSC models (MES28, GSC23, 387, 3691) generously donated by Dr. Jeremy N. Rich’s laboratory (University of Pittsburgh Medical Center, Pittsburgh, PA, USA), and neural stem cell lines (NSC11, ENSA, HNP1). All stem cell populations were maintained in serum-free DMEM/F12 medium (Gibco, Waltham, MA, USA) enriched with B27 supplement (Life Technologies, Carlsbad, CA, USA) and dual growth factors (20 ng/mL bFGF/EGF; R&D Systems, Minneapolis, MN, USA). Corresponding DGCs were propagated in DMEM basal medium (Gibco 11995065) containing 10% fetal bovine serum (Gibco 26140079) to preserve their differentiated phenotype.

### 2.6. SiRNA Transfection

ZEB1 siRNAs were purchased from Dharmacon (ON-TARGETplus Human ZEB1 siRNA, J-006564-10-0050, J-006564-11-0050). MMP9 siRNAs were purchased from Dharmacon (ON-TARGETplus Human MMP9 siRNA, J-005970-07-0050, J-005970-08-0050). ZO-1(TJP1) siRNAs were purchased from Dharmacon (ON-TARGETplus Human TJP1 siRNA, J-007746-05-0020, J-007746-06-0020). A total of 2,500,000 of GSC23 cells were electroporated with either control, ZEB1, MMP9, or ZO-1(TJP1) siRNA (100 nM) using nucleofector (kit C and program X-05). Then, the cells were plated in 5 mL of complete neurobasal media and incubated for 48 h at 37 °C until experiment analysis.

### 2.7. Immunoblotting

Protein extraction was performed with RIPA lysis buffer (P0013B, Beyotime, Shanghai, China) supplemented with protease inhibitors, and protein concentrations were determined using a BCA assay (Thermo Fisher Scientific, Waltham, MA, USA). Equal protein quantities from cellular or tissue lysates were separated on 10–17% SDS-PAGE gels. Following electrophoretic separation and membrane transfer, blots were probed with specific primary antibodies followed by Goat anti-Mouse or Goat anti-Rabbit IgG (H+L) HRP-linked secondary antibodies (31430 and 31460, Invitrogen, Waltham, MA, USA). Protein signals were detected by enhanced chemiluminescence (Clarity Western ECL Substrate, Bio-Rad, Hercules, CA, USA) and quantified through grayscale densitometry. A detailed list of primary antibodies is presented in Table 2. All the uncropped original blots are provided in Supplementary Figures S1 and S2.

### 2.8. Statistical Analysis

The statistical software R (version 4.0.3) and GraphPad Prism 10 software (version 10.1.0) were used for the statistical analysis and the generation of figures. The median values of EMT-related gene expression were considered the cutoff value to separate patients into the high and low groups. Survival analysis was conducted using Kaplan–Meier analysis and the log-rank test. The Wilcoxon signed rank test, Mann–Whitney test, and unpaired *t*-test were used for statistical analysis between two groups, while the Kruskal–Wallis test was applied for statistical analysis between more than two groups. The non-parametric Spearman test was conducted to evaluate the correlation of two variables. All statistical tests were independently performed by two different scientists, and a *p*-value of less than 0.05 was considered statistically significant.

## 3. Results

### 3.1. Initial Clustering and Identification of Cell Types in GBM

**Figure 1:**
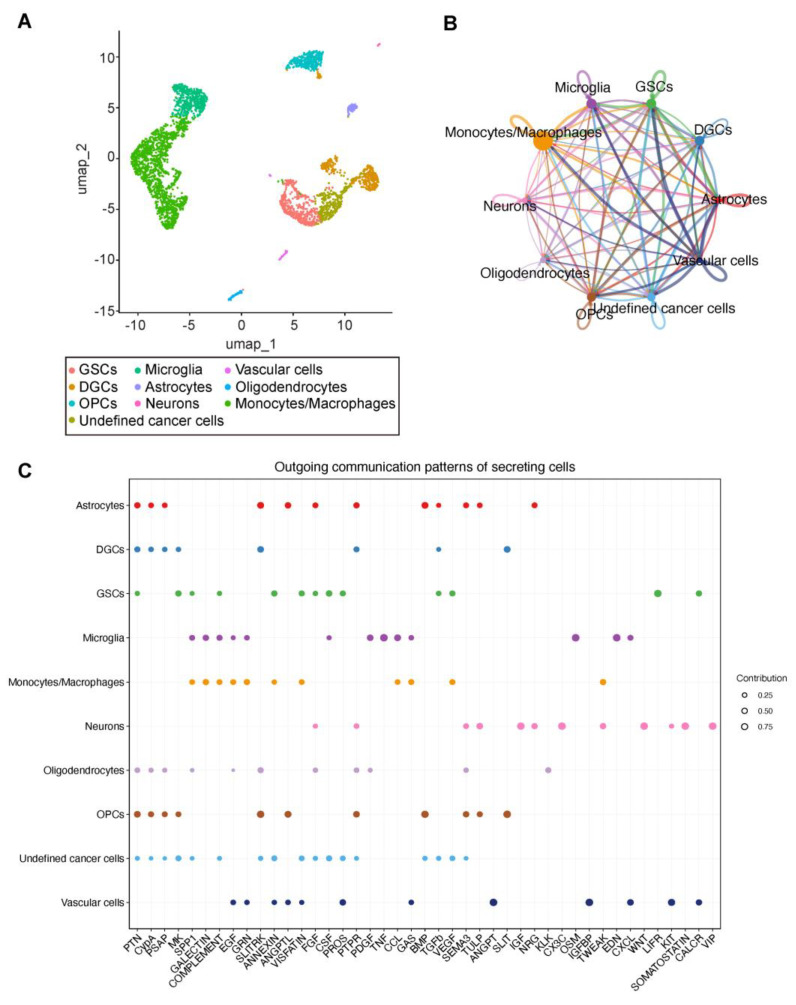
Identification of cell types and overview of the communication network in GBM. (**A**) 2D-UMAP representation of all single cells included in the study (*n* = 3589). Cell clusters are differentially colored and identified as distinct cell classes. (**B**) Cellular communication visualization of all 10 cell types. Different colors in the circle plot represent different cell groups. The interaction strength is quantified based on ligand–receptor interaction probabilities. The communication probability here only represents the interaction strength and is not an exact probability [20]. (**C**) All the outgoing signaling pathways of secreting cells. The dot color and size represent the cell group and contribution factor. Data sourced from scRNA-seq dataset (GSE84465) from the GEO database.

To investigate cell–cell communication patterns of GSCs in GBM, we analyzed a scRNA-seq dataset (GSE84465) from the GEO database containing 3589 cells derived from human primary GBM samples. We used k-means clustering on the 2D UMAP map, resulting in the identification of 10 distinct cell types within separate clusters, which includes GSCs, DGCs, oligodendrocyte precursor cells (OPCs), microglia, astrocytes, neurons, vascular cells, oligodendrocyte, monocytes/macrophages, and undefined cancer cells (Figure 1A).

### 3.2. Overview of the Communication Network in GBM

To infer and visualize intercellular communications in GBM, we ran CellChat analysis on the scRNA-seq dataset mentioned above. CellChat analysis revealed intricate cell–cell communication networks in GBM, with all pairwise combinations among the 10 cell clusters exhibiting varying degrees of interaction intensity, including notable autocrine signaling within individual cell populations (Figure 1B).

A total of 43 significant signaling pathways among the 10 cell groups were detected (Figure 1C). The signaling pathways associated with GSCs encompassed multiple molecular systems, including pleiotrophin (PTN), midkine (MK), secreted phosphoprotein 1 (SPP1), complement signaling, annexin-mediated pathways, visfatin-related mechanisms, fibroblast growth factor (FGF) signaling, colony-stimulating factor (CSF) pathways, prosaposin (PROS)-associated signaling, transforming growth factor beta (TGF-β) pathways, vascular endothelial growth factor (VEGF) signaling, leukemia inhibitory factor receptor (LIFR)-mediated communication, and calcitonin receptor (CALCR)-related interactions.

### 3.3. Cell–Cell Communication Strength Across 10 Cell Groups

**Figure 2:**
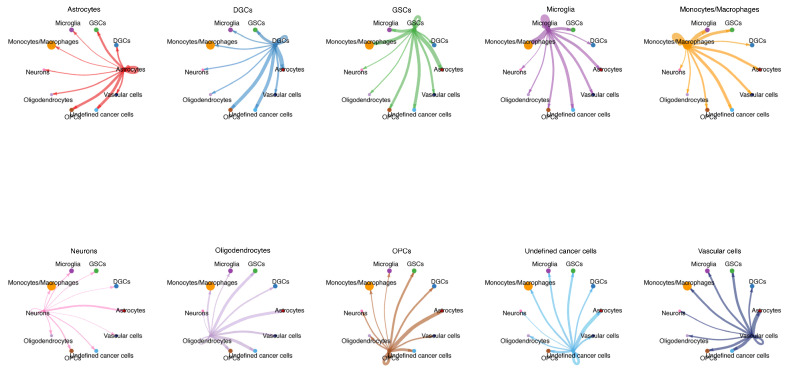
Cell–cell communication network analysis across 10 cell groups. Different colors of circles represent different cell groups.

Figure 2 illustrates the communication strength between each cell type and others, where GSCs demonstrate robust autocrine signaling (self-communication), followed by notable paracrine interactions with microglia, monocytes/macrophages, and astrocytes. Surprisingly, their communication intensities with neurons, oligodendrocytes, and DGCs were comparatively weaker.

### 3.4. The Cell Communication Pattern of GSCs Is Mostly Related to EMT

**Figure 3:**
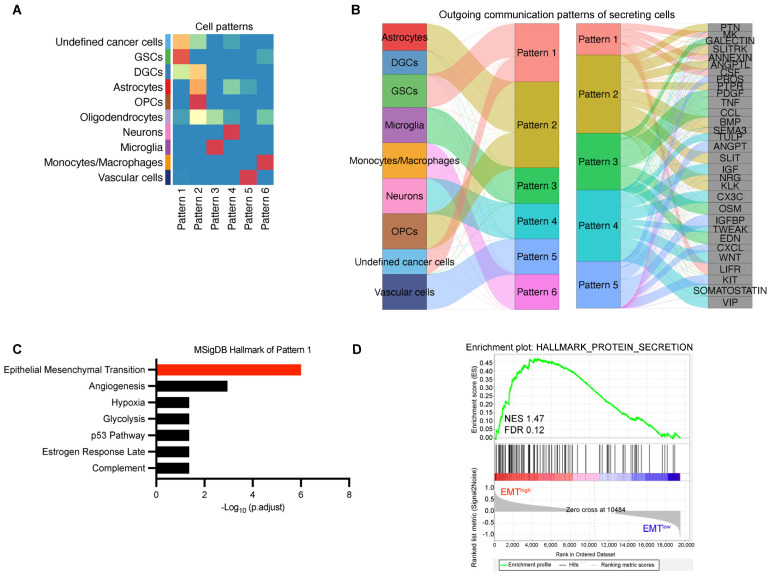
The cell communication pattern of GSCs is mostly related to EMT. (**A**) Identification of outgoing communication patterns in secretory cells. The color of the box indicates the contribution of the cell group or signaling pathway to each latent pattern, with warmer (red-shifted) spectra indicating elevated contribution indices and cooler (blue-shifted) spectra corresponding to diminished contribution values. (**B**) The inferred outgoing communication patterns of secreting cells, which shows the correspondence between the inferred latent patterns and cell groups, as well as signaling pathways. The thickness of the flow indicates the contribution of the cell group or signaling pathway to each latent pattern. (**C**) KEGG analysis of the genes associated with pathways in Pattern 1, ranked on the basis of adjusted *p*-values. (**D**) GSEA of TCGA-GBM data. Normalized enrichment score (NES) and false discovery rate (FDR) are shown.

To further investigate how multiple cellular groups and signaling pathways coordinate their functions, we employed CellChat to identify global communication patterns using a pattern recognition approach. Through the selectK function, we inferred the optimal number of communication patterns to be six. The outgoing communication patterns revealed how sender cells (i.e., signal-originating cells) coordinate with each other and interact with specific signaling pathways to drive cellular communication. Among these six outgoing patterns, GSCs were predominantly associated with Pattern 1 (Figure 3A), which encompasses signaling pathways including MK, ANNEXIN, CSF, and LIFR (Figure 3B).

KEGG analysis of the genes associated with these pathways demonstrated significant enrichment in EMT processes (Figure 3C). Previous studies have demonstrated that MK promotes EMT-mediated cisplatin resistance [33]. Members of the ANNEXIN family, including ANXA2, can form complexes with TTK, which activate the Akt/mTOR signaling pathway and promote EMT [34]. G-CSF and CSF-1 were key factors, which promoted the EMT, migration, and invasion of trophoblasts via activating the PI3K/Akt/Erk1/2 signaling pathway [35,36]. LIFR, functioning as a target of KMT2D, epigenetically activates the PI3K/Akt pathway and drives EMT [37]. Subsequent GSEA of TCGA-GBM data further revealed that enhanced enrichment of EMT-related genes correlated with increased secretory protein production capacity (Figure 3D). These findings collectively suggest a strong association between secretory protein signaling in GSCs and EMT activation.

### 3.5. Spatial Co-Localization of EMT and GSCs in GBM

**Figure 4:**
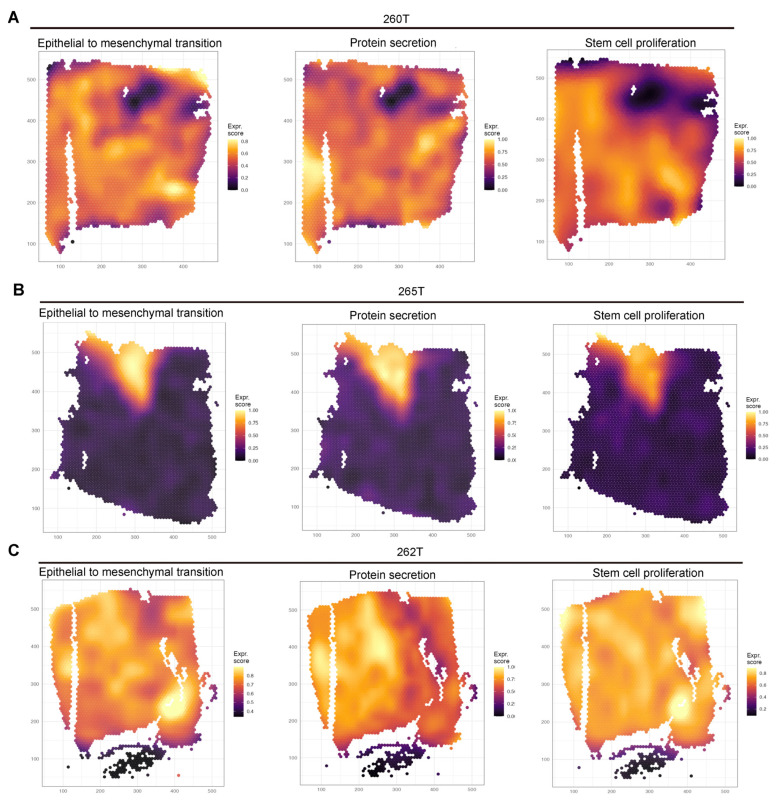
Spatial co-localization of EMT and GSCs in GBM. (**A**–**C**) Spatial GBM patient sample (UKF#260, #265, #262). Reference mapping was performed using SpaceXR (Version 1.0) and SPATA2 (Version 3.1.3) software. Data sourced from Datadryad (https://doi.org/10.5061/dryad.h70rxwdmj).

To investigate the spatial correlation between EMT expression patterns and GSCs, we analyzed spatial transcriptomic data from three GBM specimens [32] (Figure 4A–C). Intriguingly, our findings revealed striking spatial concordance among stem cell proliferation, protein secretion activity, and EMT activation patterns.

### 3.6. Elevated Expression of EMT-Associated Genes in GBM

**Figure 5:**
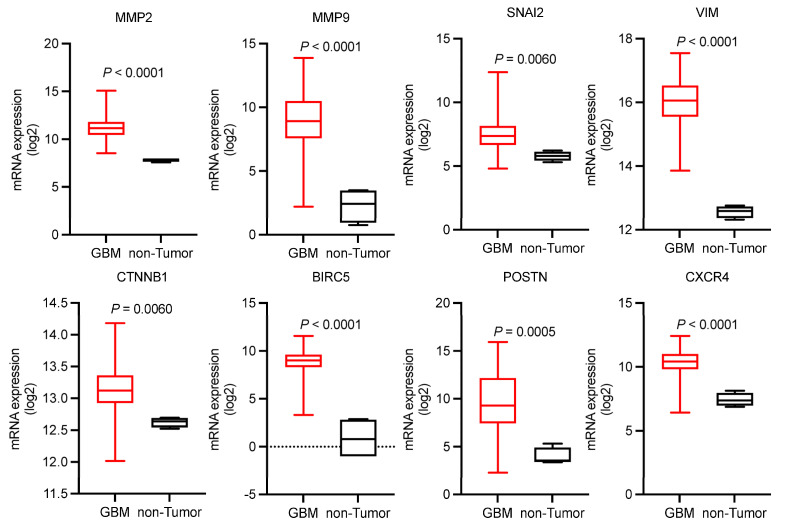
Expression profiles of 8 EMT-related genes in GBM and non-tumor tissues. Box plots represent median expression values with interquartile ranges (IQRs). Data sourced from the Gliovis platform within The Cancer Genome Atlas (TCGA) database.

Subsequently, we sought to investigate the expression patterns of EMT-related genes in GBM. Given the extensive number of EMT-associated genes, we selected eight representative genes (*MMP2*, *MMP9*, *SNAI2*, *VIM*, *CTNNB1*, *BIRC5*, *POSTN*, *CXCR4*) for analysis [38]. Their expression profiles were analyzed using Gliovis platform within The Cancer Genome Atlas (TCGA) database. Comparative analysis revealed significant upregulation of all eight examined genes in GBM tissues compared to non-tumor controls (Figure 5). The majority of EMT-related genes in Pattern 1 of Figure 3C were also highly expressed in TCGA-GBM (Supplementary Figure S3).

### 3.7. Elevated Expression of EMT-Associated Genes Correlates with Poor Prognosis

**Figure 6:**
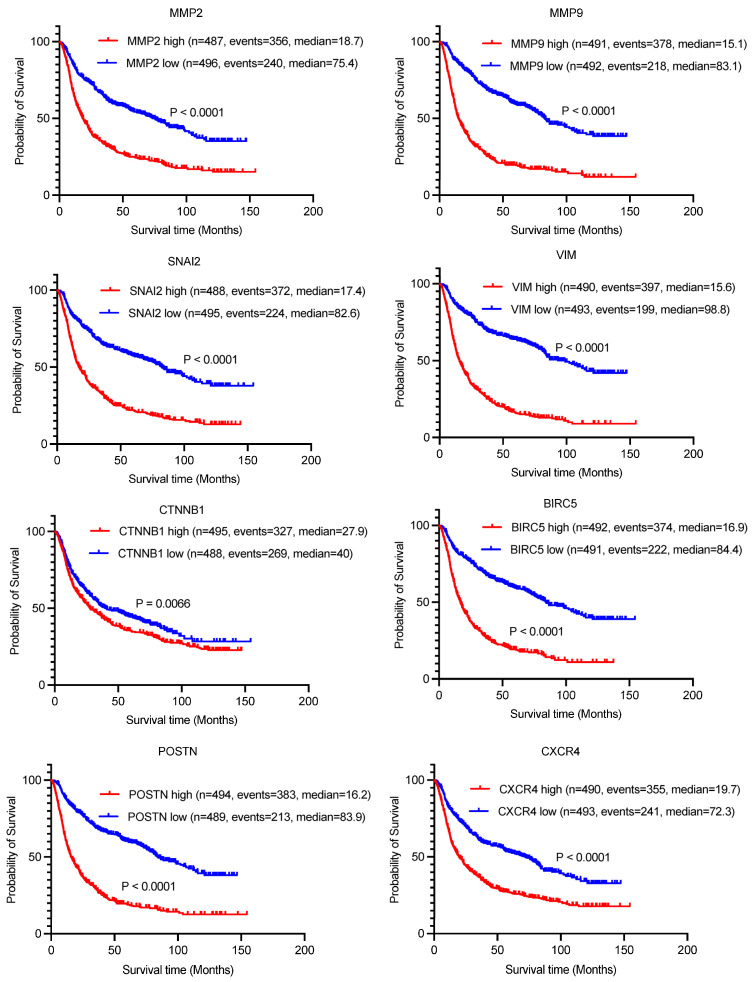
Kaplan–Meier survival curves illustrating the association between the expression levels of 8 EMT-related genes and overall survival in glioma patients. Patients were stratified into high-expression (red lines) and low-expression (blue lines) groups based on median gene expression values. Log-rank test *p*-values are displayed for each gene. Data sourced from CGGA glioma cohort.

Next, we investigated the prognostic relevance of these highly expressed genes in glioma patients using the Chinese Glioma Genome Atlas (CGGA) database. Our analysis revealed that all eight genes exhibited significantly shorter overall survival times in high-expression groups compared to their low-expression counterparts, suggesting a negative correlation between these genes and patient prognosis (Figure 6).

### 3.8. GSCs Exhibit Pronounced Overexpression of EMT-Associated Gene Signature in GBM

**Figure 7:**
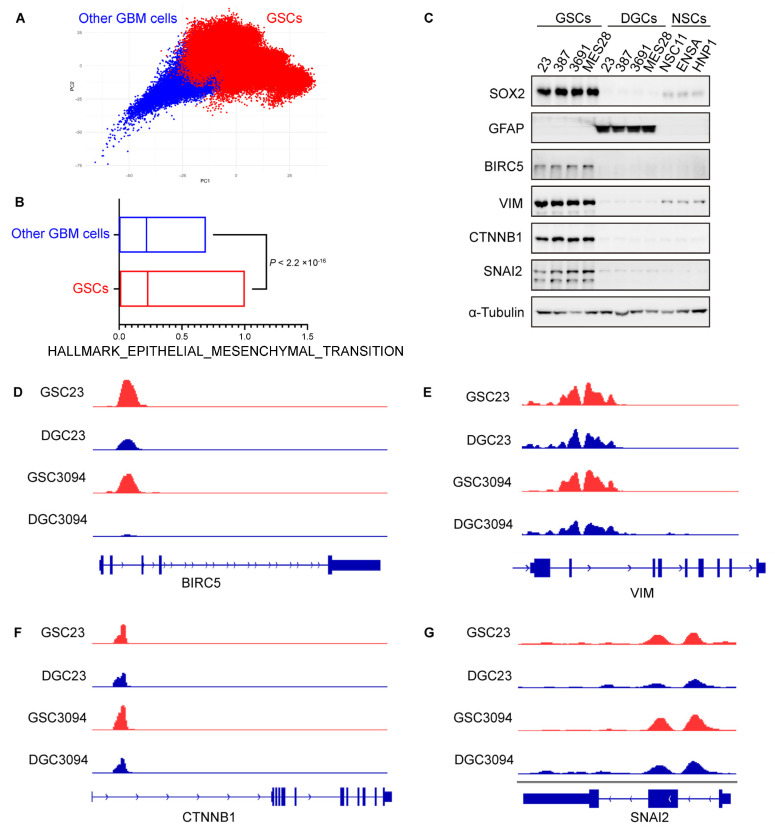
GSCs exhibit pronounced overexpression of EMT-associated gene signature in GBM. (**A**) Distribution of all GSCs (*n* = 65,655), including 28 early-passage GSC cultures derived from 24 patients and all tumor cells (*n* = 14,207) from seven patients with GBM. (**B**) Quantification of hallmark of EMT in scRNA-seq data. Violin plots represent the overall distribution of data points. Box plots show median, upper, and lower quartiles; whiskers depict 1.5 times the interquartile range. (**A**,**B**) Data sourced from the Broad Institute Single-Cell Portal (https://singlecell.broadinstitute.org/single_cell/study/SCP503, accessed on 17 March 2025) and CReSCENT60 (https://crescent.cloud; accessed on 16 March 2025, study ID CRES-P23). (**C**) Immunoblots showing BIRC5, VIM, CTNNB1, and SNAI2 expression in the indicated cells. GFAP is a differentiation marker, and SOX2 is a stem cell marker. Immunoblots are representative of three independent experiments with similar results. (**D**–**G**) H3K27ac ChIP-seq tracks at BIRC5, VIM, CTNNB1, and SNAI2 gene loci. (**D**–**G**) Data sourced from the GEO dataset (GSE129438).

To compare the expression of EMT-related genes between GSCs and other GBM cells, we analyzed an additional scRNA-seq dataset comprising 28 early-passage GSC cultures derived from 24 patients and 14,207 malignant cells from seven patients with GBM [39] (Figure 7A). Our analysis revealed significantly higher expression levels of EMT-associated genes in GSCs compared to other GBM cell populations (Figure 7B).

The protein expression levels of BIRC5, VIM, CTNNB1, and SNAI2 were demonstrated by immunoblot analysis to be significantly elevated in four GSC lines (MES28, GSC23, 387, 3691) compared to their DGC counterparts and three NSC lines (NSC11, ENSA, HNP1) (Figure 7C). In two pairs of matched GSCs and DGCs analyzed by H3K27ac ChIP-seq, the H3K27ac peaks of BIRC5, VIM, CTNNB1, and SNAI2 were consistently elevated in GSCs compared to their corresponding DGC counterparts (Figure 7D–G). This observation suggests that EMT-associated genes exhibit enhanced expression in GBM, particularly within the GSC population.

### 3.9. EMT-Related Genes Are More Enriched in MES-like GSCs

**Figure 8:**
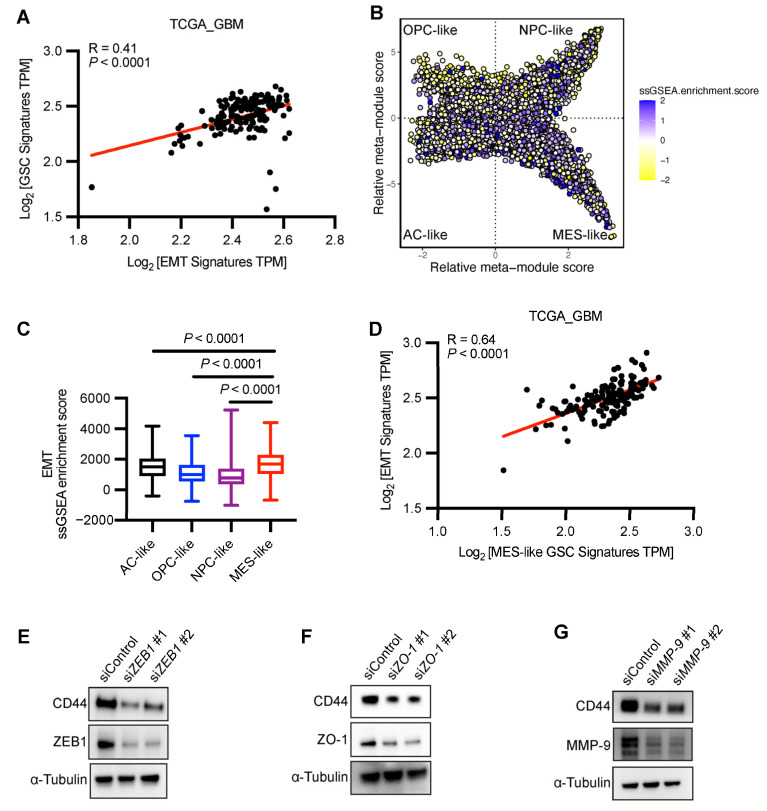
EMT-related genes are more enriched in MES-like GSCs. (**A**) Pearson correlation of EMT signatures and GSC signatures in RNA-seq data of TGGA_GBM. The red line shows linear regression. (**B**) Two-dimensional representation of cellular states. Each quadrant corresponds to one cellular state, the exact position of malignant cells (dots) reflects their relative scores for the meta-modules, and their colors reflect the ssGSEA enrichment scores of EMT. (**C**) Box plots depict ssGSEA enrichment scores of EMT-related gene signatures across four GSC states. Each box represents the distribution of scores for a specific GSC state: AC-like (black), OPC-like (blue), NPC-like (purple), and MES-like (red). The central line within each box indicates the median value, while the box spans the interquartile range (IQR). Whiskers extend to 1.5× IQR. Statistical significance between groups was calculated by Wilcoxon rank-sum test. (**B**,**C**) Data sourced from GSE131928. (**D**) Pearson correlation of EMT signatures and MES-like GSC signatures in RNA-seq data of TGGA_GBM. The red line shows linear regression. (**E**–**G**) GSC23 cells were transfected with control, *ZEB1*, *ZO-1*, or *MMP-9* siRNAs for 48 h. Then, immunoblots were performed using CD44, ZEB1, ZO-1, MMP-9, and α-tubulin antibodies.

As previously demonstrated, a subset of EMT-related genes exhibits elevated expression in GSCs, and the EMT signatures demonstrate a positive correlation with the GSC signatures (Figure 8A).

It was reported that different states of GSCs that are thought to act as key cancer drivers can spread tumors and resist radiation/chemo treatments better than other cells [8,40]. Cyril Neftel and colleagues categorized GSCs into four distinct phenotypic states based on cellular markers [14]: OPC-like, NPC-like, AC-like, and MES-like, which demonstrates the diversity of proliferating cells. To investigate the association between EMT-related genes and different GSC states, we performed ssGSEA analysis on those four GSC subtypes (GSE131928). The results revealed that MES-like GSCs exhibited the highest enrichment scores for EMT-related genes (Figure 8B,C), indicating their strongest correlation with the mesenchymal state. In the TCGA-GBM database, EMT-related genes also demonstrated significant correlations with MES-like GSC signatures (Figure 8D).

To confirm that EMT activation precedes MES differentiation in GBM progression, we transfected GSC23 cells with siRNAs targeting the most common EMT markers, e.g., *ZEB1*, *ZO-1*, and *MMP-9*, and subsequently examined the protein levels of CD44, a marker of the mesenchymal GSC subtype (Figure 8E–G). The results demonstrated that knockdown of EMT-related genes significantly reduced CD44 levels, further confirming that EMT activation precedes mesenchymal differentiation.

### 3.10. Clinical Significance: Worse Prognosis Associated with High MES-like Signature Expression in Brain Tumor Patients

**Figure 9:**
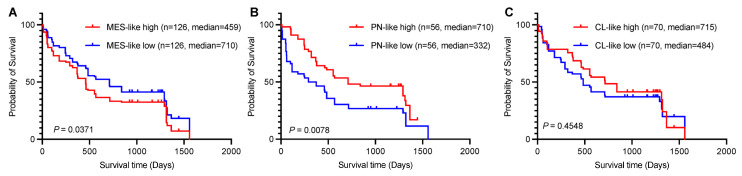
High MES-like signature expression is associated with poor prognosis in brain tumor patients. (**A**–**C**) Kaplan–Meier survival curves illustrating the association between the expression levels of MES-like, PN-like, CL-like, and overall survival in all tumor patients. Patients were stratified into high-expression (red lines) and low-expression (blue lines) groups based on median gene expression values. Log-rank test *p*-values are displayed for each gene. Data sourced from the Brain TIME platform [43].

Given the close association between EMT-related genes and MES-like GSCs, we next sought to investigate the clinical significance of MES-like signatures. Studies of intertumoral heterogeneity based on bulk expression profiles suggest that three subtypes of GBM exist, namely proneural (PN), classical (CL), and mesenchymal (MES) [41,42]. We performed survival analyses on all brain tumor patients [43] using the Brain TIME platform with these three subtypes of signatures (Figure 9A–C) and found that only the MES-like group demonstrated a significantly worse prognosis when exhibiting high expression levels (*p* = 0.0371).

## 4. Discussion

This study explored the communication pattern of GSCs in GBM. Existing studies have investigated cell–cell communication in GBM [44,45,46,47,48]; however, the majority have predominantly focused on elucidating the interactions between GSCs and immune cell populations [48,49]. For example, Cao et al. found that the MIF pathway was active between GSCs and myeloid-derived suppressor cells (MDSCs) [48]. GSCs may activate MDSCs to inhibit immune responses by secreting MIF. Li et al. identified important ligand–receptor interactions, including MIF binding with CD74 + CXCR4 or CD74 + CD44 and SPP1 binding with CD44 or ITGAV + ITGB1 [49]. They also highlighted the key role and higher interaction between monocytes/macrophages and MES-like malignant cells in the SPP1 signaling pathway, which is consistent with our results (Figure 1C). In our study, we focused on the most relevant biological functions of GSC signal pathways after enrichment analysis. Through KEGG analysis, we found that the cell communication patterns of GSC are most closely associated with EMT, suggesting that GSCs may play a role in promoting the mesenchymal transition of GBM (Figure 3).

The process of EMT involves a series of biochemical transformations in which epithelial cells, originally organized in a polarized sheet, acquire mesenchymal characteristics. This transition results in cells that exhibit reduced intercellular adhesion, increased mobility, and a mesenchymal phenotype. Additionally, these transformed cells develop distinct morphological features, enhanced resistance to apoptosis induced by detachment (anoikis), and increased tolerance to chemotherapy [50,51].

Because GBMs are not typical epithelial cells due to the absence of a basal membrane and inconsistent expression of E-cadherin, to coin the term “EMT” within the GBM context would be scientifically inaccurate. However, studies in recent years have found that the regulation of the classical EMT markers can induce the GBM cells to the invasive MES subtype [52,53]. Compared to bulk RNA-seq approaches in prior studies by Neftel et al. [14], we used CellChat to infer cell–cell communication and revealed that the expression of EMT-associated genes may serve as a driving force for MES-like phenotypic transformation, suggesting that these genes may act as transcriptional regulators of the MES phenotype. Our findings also indicate that EMT-related genes are more enriched in MES-like GSCs (Figure 8). These findings highlight the parallels in genetic profiles shared by both the MES transition and the classical EMT process, encompassing key elements such as traditional EMT markers, transcription factors, and the activation of signaling pathways.

Our study also revealed that MES signatures are associated with poorer prognostic outcomes in brain tumor patients compared with PN or CL signatures (Figure 9A). The PN and MES expression subtypes have been most consistently described in the literature, with PN relating to a more favorable outcome and MES relating to poor survival [54,55,56], which is consistent with our results with different datasets (Figure 9A,B). MES-like glioma cell differentiation status has been found to correlate with enrichment of macrophages/microglia [41,57,58]. It is also reported that EMT is the main way in which glioma-associated microglia/macrophages (GAMs) promote glioma progression [59]. These findings suggest that GSCs may facilitate MES differentiation through EMT-associated cell communication patterns, subsequently recruiting immunosuppressive GAMs. This cascade could contribute to GBM immune evasion, thereby promoting tumor progression. However, further experimental validation is required to substantiate these conclusions.

This study also has certain limitations and shortcomings. Firstly, the scRNA-seq database we utilized for CellChat contained a relatively small number of cells (*n* = 3589). To further validate our conclusions, it will be necessary to employ larger databases with greater cell numbers in future studies. Second, we relied on published retrospective datasets, necessitating future validation in a prospective, large cohort. Third, we should conduct more in-depth and detailed molecular biology studies in both in vivo and in vitro experiments to uncover the molecular mechanisms of MES differentiation of GSCs and identify new therapeutic targets.

## 5. Conclusions

Our study suggests that GSCs facilitate GBM progression through intercellular communication in the pattern of EMT. EMT-associated genes may drive the differentiation of GSCs toward a MES-like phenotype, thereby leading to poorer clinical outcomes. Consequently, targeting EMT-related pathways could represent a novel therapeutic strategy for GBM treatment.

## Figures and Tables

**Table 1:** Detailed information on the datasets used in this study.

Data	Species	GPL	Data Type	Information
GSE84465	Homo sapiens	GPL18573	scRNA-seq	Examination of cell types in human GBM samples
GSE129438	Homo sapiens	GPL11154	CHIP-seq	H3K27ac ChIP-seq of 2 pairs of GSCs and matched DGCs
GSE131928	Homo sapiens	GPL18573	scRNA-seq	24,131 single cells from 28 patients with GBM

**Table 2:** Key resources.

Antibodies	Source	Cat No.
SOX2 polyclonal antibody	Proteintech	11064-1-AP
GFAP polyclonal antibody	Proteintech	16825-1-AP
SURVIVIN (BIRC5) polyclonal antibody	Proteintech	10508-1-AP
Vimentin (VIM) polyclonal antibody	Proteintech	10366-1-AP
Beta catenin polyclonal antibody	Proteintech	51067-2-AP
SNAI2/SLUG polyclonal antibody	Proteintech	12129-1-AP
Alpha tubulin polyclonal antibody	Proteintech	11224-1-AP
CD44 polyclonal antibody	Proteintech	15675-1-AP
ZEB1 polyclonal antibody	Proteintech	21544-1-AP
ZO-1 polyclonal antibody	Proteintech	21773-1-AP
MMP-9 (N-terminal) polyclonal antibody	Proteintech	10375-2-AP

## Data Availability

All data can be obtained from the corresponding author.

## Supplementary Materials

The following supporting information can be downloaded at: https://www.mdpi.com/article/10.3390/biomedicines13061304/s1, Figure S1: Original images for immunoblots in Figure 7C; Figure S2: Original images for immunoblots in Figure 8E–G; Figure S3: Expression profiles of EMT-related genes in Pattern 1 of Figure 3C in TCGA-GBM and non-tumor tissues.

## Abbreviations

The following abbreviations are used in this manuscript:

GBM	Glioblastoma
CNS	Central nervous system
SoC	Standard of care
GSCs	Glioma stem cells
TME	Tumor microenvironment
scRNA-seq	Single-cell RNA sequencing
EMT	Epithelial–mesenchymal transition
GEO	Gene Expression Omnibus
NCBI	National Center for Biotechnology Information
PCA	Principal component analysis
DGCs	Differentiated glioma cells
IGV	Integrative Genomics Viewer
KEGG	Kyoto Encyclopedia of Genes and Genomes
GSEA	Gene set enrichment analysis
ssGSEA	Single-sample GSEA
OPCs	Oligodendrocyte precursor cells
PTN	Pleiotrophin
MK	Midkine
SPP1	Secreted phosphoprotein 1
FGF	Fibroblast growth factor
CSF	Colony-stimulating factor
PROS	Prosaposin
TGF-β	Transforming growth factor beta
VEGF	Vascular endothelial growth factor
LIFR	Leukemia inhibitory factor receptor
CALCR	Calcitonin receptor
NES	Normalized enrichment score
FDR	False discovery rate
TCGA	The Cancer Genome Atlas
CGGA	Chinese Glioma Genome Atlas
OPC-like	Oligodendrocyte precursor cell-like
NPC-like	Neural progenitor cell-like
AC-like	Astrocyte-like
MES-like	Mesenchymal-like
IQR	Interquartile range
PN	Proneural
CL	Classical
MDSCs	Myeloid-derived suppressor cells
GAMs	Glioma-associated microglia/macrophages
