# Supplementary material for: Multi-Transcriptomic Analysis Reveals GSC-Driven MES-Like Differentiation via EMT in GBM Cell–Cell Communication
Source: Biomedicines. 2025 May 26;13(6):1304. doi: 10.3390/biomedicines13061304 (PMC12189402; doi:10.3390/biomedicines13061304)

Figure S3. Expression profiles of EMT-related genes in Pattern 1 of Figure 3C in TCGA-GBM and non-tumor tissues.

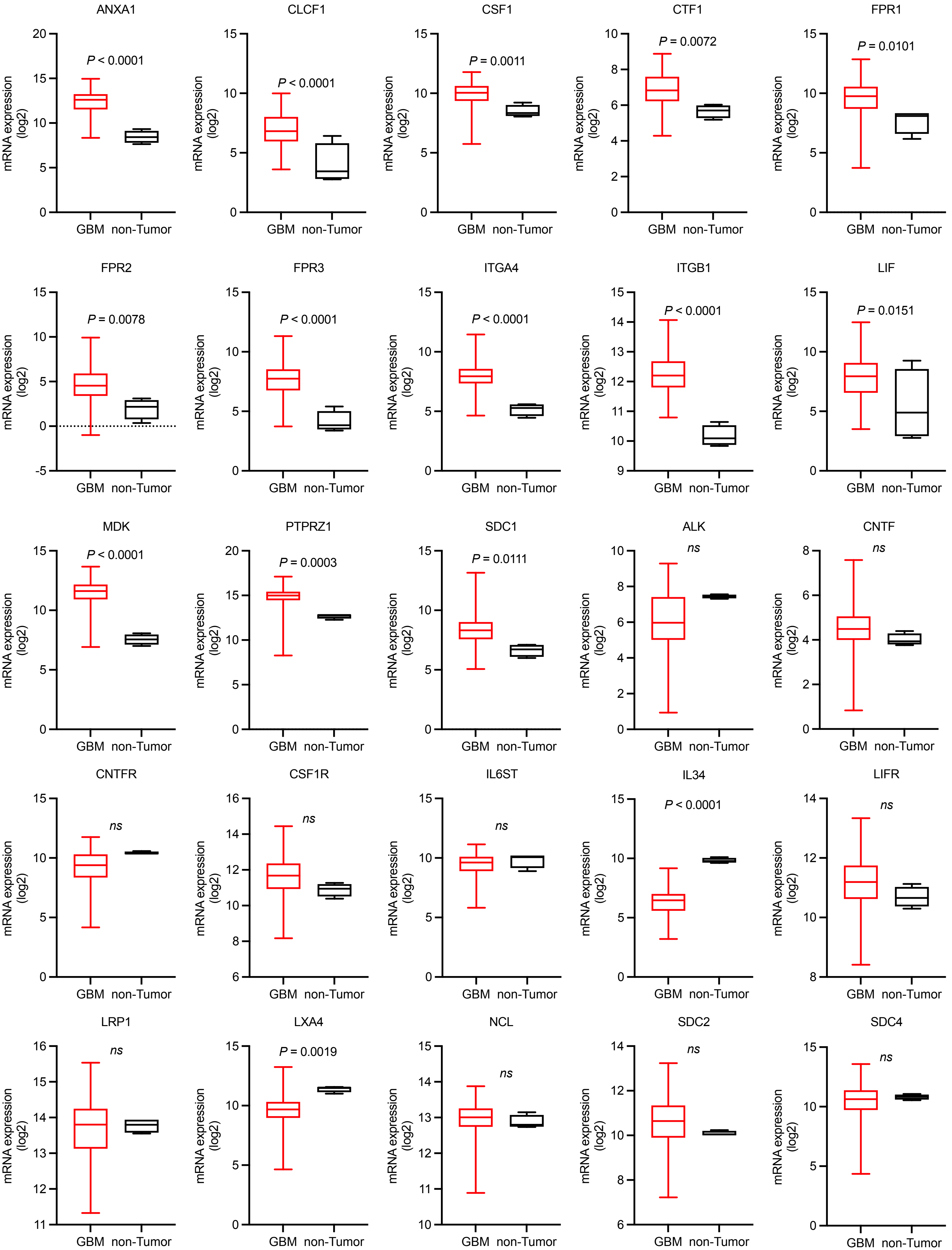

Supplement: Supplementary file 1 [file biomedicines-13-01304-s001.zip › Figure S3.pdf]
