# Supplementary figures and images for: Multi-Transcriptomic Analysis Reveals GSC-Driven MES-Like Differentiation via EMT in GBM Cell–Cell Communication
Source: Biomedicines. 2025 May 26;13(6):1304. doi: 10.3390/biomedicines13061304 (PMC12189402; doi:10.3390/biomedicines13061304)

**Figure S2.** Original images for immunoblots in Figure 8E-G.

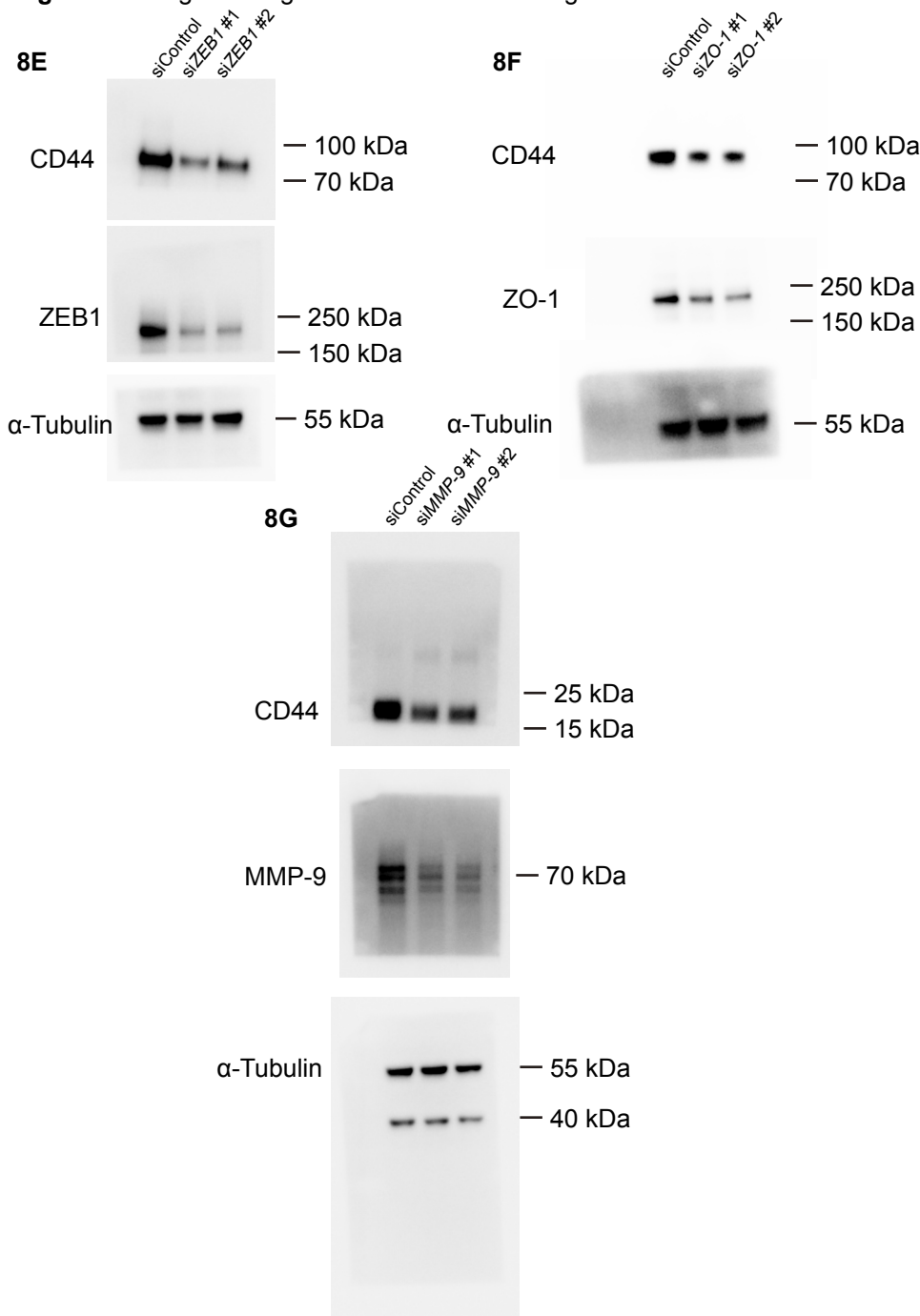

Supplement: Supplementary file 1 [file biomedicines-13-01304-s001.zip › Figure S2.pdf]

**Figure S1.** Original images for immunoblots in Figure 7C.

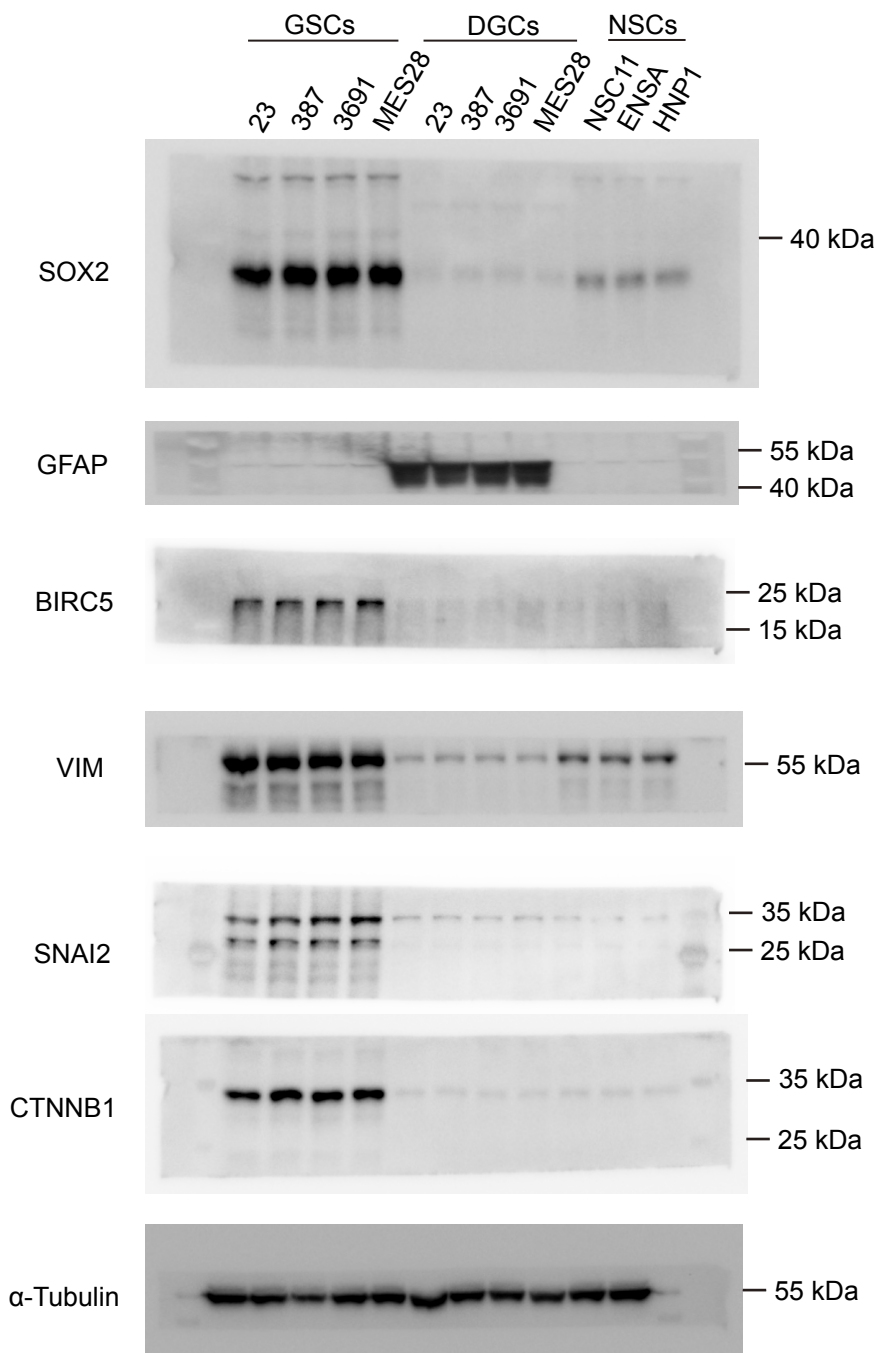

Supplement: Supplementary file 1 [file biomedicines-13-01304-s001.zip › Figure S1.pdf]
